# Design and Rationale of Prolonged Nightly Fasting for Multiple Myeloma Prevention (PROFAST): Protocol for a Randomized Controlled Pilot Trial

**DOI:** 10.2196/51368

**Published:** 2024-03-11

**Authors:** David J Lee, Elizabeth K O'Donnell, Noopur Raje, Cristina Panaroni, Robert Redd, Jennifer Ligibel, Dorothy D Sears, Omar Nadeem, Irene M Ghobrial, Catherine R Marinac

**Affiliations:** 1 Department of Medicine Massachusetts General Hospital Boston, MA United States; 2 Department of Medical Oncology Dana-Farber Cancer Institute Boston, MA United States; 3 Harvard Medical School Boston, MA United States; 4 Center for Early Detection and Interception of Blood Cancers Dana-Farber Cancer Institute Boston, MA United States; 5 Biostatistics and Computational Biology Dana-Farber Cancer Institute Boston, MA United States; 6 College of Health Solutions Arizona State University Phoenix, AZ United States

**Keywords:** MGUS, smoldering myeloma, cancer prevention, intermittent fasting, fasting, myeloma, cancer, oncology, oncological, overweight, weight, obese, obesity, tumor, tumors, RCT, randomized, controlled trial, controlled trials, body mass index, BMI, blood, hematology, hematological, gammopathy, eating, diet, dietary

## Abstract

**Background:**

Obesity is an established, modifiable risk factor of multiple myeloma (MM); yet, no lifestyle interventions are routinely recommended for patients with overweight or obesity with MM precursor conditions. Prolonged nightly fasting is a simple, practical dietary regimen supported by research, suggesting that the synchronization of feeding-fasting timing with sleep-wake cycles favorably affects metabolic pathways implicated in MM. We describe the design and rationale of a randomized controlled pilot trial evaluating the efficacy of a regular, prolonged nighttime fasting schedule among individuals with overweight or obesity at high risk for developing MM or a related lymphoid malignancy.

**Objective:**

We aim to investigate the effects of 4-month prolonged nightly fasting on body composition and tumor biomarkers among individuals with overweight or obesity with monoclonal gammopathy of undetermined significance (MGUS), smoldering multiple myeloma (SMM), or smoldering Waldenström macroglobulinemia (SWM).

**Methods:**

Individuals with MGUS, SMM, or SWM aged ≥18 years and a BMI of ≥25 kg/m^2^ are randomized to either a 14-hour nighttime fasting intervention or a healthy lifestyle education control group. Participants’ baseline diet and lifestyle patterns are characterized through two 24-hour dietary recalls: questionnaires querying demographic, comorbidity, lifestyle, and quality-of-life information; and wrist actigraphy measurements for 7 days. Fasting intervention participants are supported through one-on-one telephone counseling by a health coach and automated SMS text messaging to support fasting goals. Primary end points of body composition, including visceral and subcutaneous fat (by dual-energy x-ray absorptiometry); bone marrow adiposity (by bone marrow histology); and tumor biomarkers, specifically M-proteins and serum free light-chain concentrations (by gel-based and serum free light-chain assays), are assessed at baseline and after the 4-month study period; changes therein from baseline are evaluated using a repeated measures mixed-effects model that accounts for the correlation between baseline and follow-up measures and is generally robust to missing data. Feasibility is assessed as participant retention (percent dropout in each arm) and percentage of days participants achieved a ≥14-hour fast.

**Results:**

The PROlonged nightly FASTing (PROFAST) study was funded in June 2022. Participant recruitment commenced in April 2023. As of July 2023, six participants consented to the study. The study is expected to be completed by April 2024, and data analysis and results are expected to be published in the first quarter of 2025.

**Conclusions:**

PROFAST serves as an important first step in exploring the premise that prolonged nightly fasting is a strategy to control obesity and obesity-related mechanisms of myelomagenesis. In evaluating the feasibility and impact of prolonged nightly fasting on body composition, bone marrow adipose tissue, and biomarkers of tumor burden, this pilot study may generate hypotheses regarding metabolic mechanisms underlying MM development and ultimately inform clinical and public health strategies for MM prevention.

**Trial Registration:**

ClinicalTrials.gov NCT05565638; http://clinicaltrials.gov/ct2/show/NCT05565638

**International Registered Report Identifier (IRRID):**

DERR1-10.2196/51368

## Introduction

Multiple myeloma (MM) is the second most common hematologic malignancy in the United States and is preceded by well-defined precursor conditions called monoclonal gammopathy of undetermined significance (MGUS) and smoldering MM (SMM) [1,2]. MGUS and SMM are asymptomatic conditions that have an annual progression rate to overt MM of 1% and 10%, respectively, but can be as high as 58% in 20 years in certain risk groups [3,4]. Despite the increasingly strong interest in intervening at the precursor stage for the early interception and prevention of MM, safe and cost-effective interventions are lacking. Although clinical trials have shown that earlier initiation of anti-MM therapies at the precursor stage (eg, lenalidomide-based regimen for high-risk SMM [5]) may alter the natural disease course and improve survival [5,6], early treatment remains highly controversial due to high costs and toxicity risks to patients, limiting its use to only a subset of patients at the highest risk strata [7-10].

Lifestyle interventions targeting excess adiposity and metabolic health in precursor patients may have an important role in MM prevention. Obesity is a well-established, potentially modifiable risk factor of MM [11], and there is accumulating evidence that obesity may also increase the risk of MGUS and its progression to overt MM [12-14]. Although current dietary and weight control guidelines for cancer prevention focus largely on calorie restriction and optimizing intake of specific food groups [15], challenges related to their integration into individuals’ daily lives for a sustained period of time remain a consideration [16-19]. Time-restricted feeding, a form of intermittent fasting whereby ad libitum energy consumption is constrained to a window of time (typically between 4 and 12 hours daily), may be a simple, feasible alternative for weight loss and cancer risk reduction [16,20]. When food intake timing occurs during the wake phase of the 24-hour day, time-restricted feeding may benefit metabolic health and cancer risk by synchronizing feeding-fasting regimens with daily circadian rhythms, which, in turn, improve oscillations in the circadian clock expression of numerous genes important for glucose metabolism and overall cellular homeostasis (eg, autophagy and DNA damage repair) [16,21,22].

The PROlonged nightly FASTing (PROFAST) study is a randomized controlled pilot trial investigating the clinical benefit of a 4-month prolonged nightly fasting regimen in individuals with overweight or obesity with MGUS, those with SMM, and those with smoldering Waldenström macroglobulinemia (SWM). The intervention is supported by evidence that prolonged nighttime fasting is not only a simple and sustainable behavior change [16,23] but also improves metabolism and body weight regulation [24-29]. Here, we describe the study design, rationale, and framework for assessing the potential clinical significance of a low-risk, cost-effective lifestyle intervention in patients with MM precursor conditions.

## Methods

### Design of PROFAST

PROFAST is a pilot randomized controlled trial of our 4-month prolonged nightly fasting intervention in patients with overweight or obesity with MGUS, SMM, or SWM. The goal of the trial is to acquire preliminary outcome data for the efficacy of prolonged nightly fasting on body composition and clinical markers of disease progression in precursor patients. Participants are randomly assigned to (1) a theory-based intervention designed to promote a 14-hour fast during the nighttime hours or (2) the healthy lifestyle education control group.

### Ethical Considerations

All study procedures and materials have been approved by the institutional review board at the Dana-Farber Cancer Institute (22-071).

### Participants and Eligibility

Eligible participants are at least 18 years old, have a BMI of ≥25 kg/m^2^, and have a documented diagnosis of MGUS, SMM, or SWM via the review of their electronic medical records. As shown in Textbox 1, inclusion criteria include individuals who (1) are currently fasting for <14 hours per night as assessed via self-report and using 24-hour food recalls and (2) own a cell phone and are capable of sending and receiving SMS text messages comfortably. Exclusion criteria are patients with (1) overt MM; (2) other cancers requiring active therapy; (3) diabetes mellitus, which may increase the risk of hypoglycemia with a prolonged fast, unless the physician who manages their clinical care provides consent that they may enroll; or (4) any other condition or circumstance that, in the investigators’ judgment, would be a contraindication to nightly fasting or interfere with trial participation (eg, night shift work, night eating syndrome, taking weight loss medication, and participation in another weight loss program). Participants are primarily recruited from the Dana-Farber Cancer Institute Center for Early Detection and Interception of Blood Cancers, a clinic focused on evaluating patients diagnosed with precursor conditions of hematologic malignancies and which works with patients to manage their risk of disease progression.

**Study eligibility criteria.**

**Inclusion criteria**

- Age ≥18 years
- BMI ≥25 kg/m^2^
- Diagnosis of monoclonal gammopathy of undetermined significance, smoldering multiple myeloma, or smoldering Waldenström macroglobulinemia
- Currently fasting for <14 hours per night, as assessed by 24-hour food recalls
- Owns a cell phone capable of sending and receiving SMS text messages comfortably
- Ability to understand and willing to sign a written informed consent document

**Exclusion criteria**

- Diagnosis of overt multiple myeloma
- Diagnosis of another malignancy requiring active therapy
- Diagnosis of diabetes mellitus, unless consent from the patient’s physician managing the participant’s clinical care
- Any medical or lifestyle condition contraindicated in or would interfere with study intervention (eg, night eating syndrome and night shift work)
- Currently taking medications intended for weight loss or participating in other weight loss programs

### Clinic Visits and Randomization

**Figure 1:**
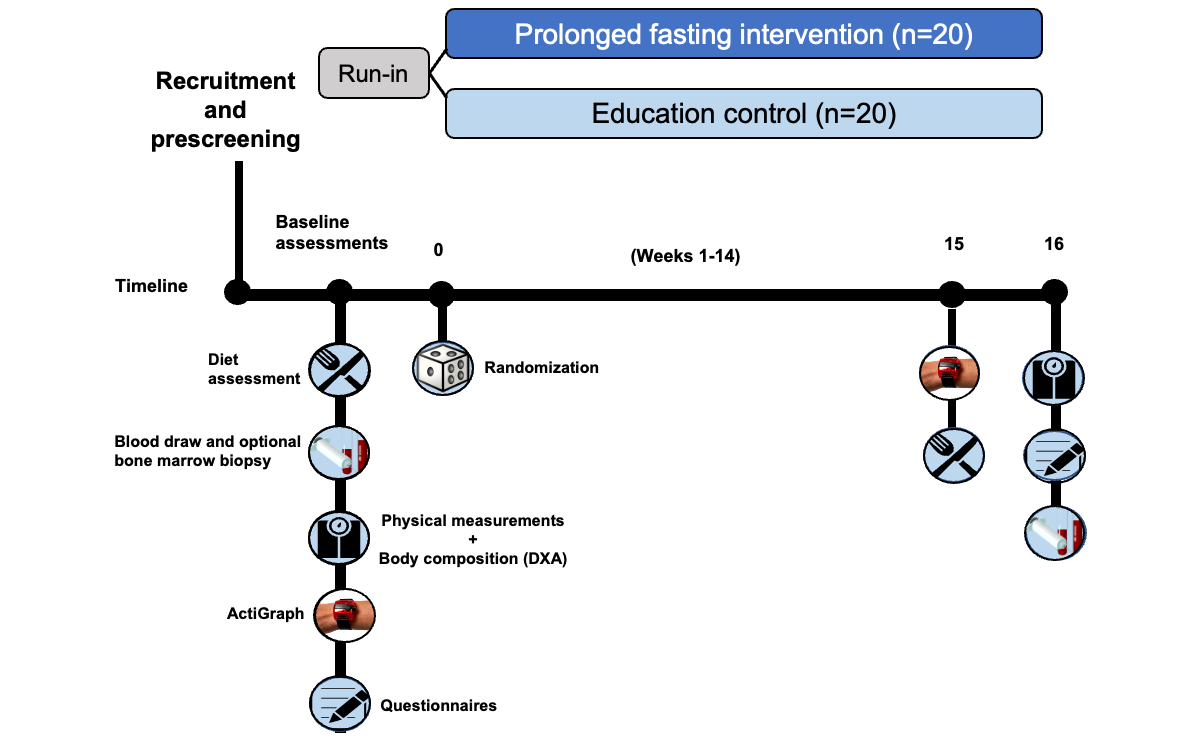
Schematic of the study design. Timeline of participant recruitment, baseline visit, randomization to intervention or control arm, and end-of-study visit, with assessments that include tumor biomarkers, body composition, 24-hour food recalls, quality-of-life and lifestyle survey, and ActiGraph.

On completion of the consent form, participants attend a baseline clinic visit that includes laboratory, self-report, and physical assessments, as shown in Figure 1. In particular, participants have their height and weight measured and receive a dual-energy x-ray absorptiometry (DXA) scan to evaluate body composition. Participants provide biospecimen samples (ie, blood and bone marrow aspirate or biopsy). For each blood draw, approximately 30 to 60 mL of blood per participant is collected into EDTA and serum separator tubes for immediate plasma and serum preparation, respectively, and aliquots of plasma and serum in 1.8-mL cryovials are placed in a –80 °C freezer for storage. As for bone marrow samples, approximately 20 mL of bone marrow is collected into EDTA tubes for immediate processing, and the processed samples are placed in cryovials in a slow freezing cryostorage for 1 to 7 days before moving to storage in a liquid nitrogen tank.

Participants’ current diet and lifestyle patterns are characterized through two 24-hour dietary recalls: (1) questionnaires ascertaining demographic, comorbidity, lifestyle, and quality-of-life information; and (2) the wearing of an ActiGraph accelerometer (GT3XP-BTLE; ActiGraph Corp) on their wrists for 7 days for baseline assessment of sleep, physical activity, and circadian rhythm.

Within 28 days of completing baseline assessments, participants are randomized in a 1:1 manner to either the nighttime 14-hour fast intervention group or the healthy lifestyle education comparison group. Once randomized, participants initiate the intervention or control condition ideally no later than 28 days of baseline screening assessments.

A clinic visit is scheduled 4 months after initiating and ideally within 7 days of completing the intervention or control condition. At this final visit, participants’ weight, biospecimens and clinical labs, DXA scan, and self-reported outcome measures are reobtained.

### Details of the Intervention

The intervention’s goal is to achieve a 14-hour nightly fast. Fasting goals during the initial weeks of the study are individualized, allowing participants to gradually adopt a 14-hour fasting duration on or before the third week of study. A 14-hour window was selected because it has been demonstrated to be an achievable intervention target in other populations of adults with chronic health conditions, and studies to date suggest that it is a sustainable intervention target [29,30]. Specifically, previous interventions have reported elective continuation of the intervention beyond the intervention period, resulting in sustained health benefits including weight loss [29,30]. The overnight fasting period is defined as the longest interval of time overnight in which no calorie-containing foods or beverages are consumed. Given prior findings that fasting may be more beneficial when aligned with the biological night [26], participants are encouraged to begin their nightly fast by 8 PM. To maximize acceptability and adherence to the intervention protocol, participants are allowed to consume noncaloric beverages including water, plain coffee, plain tea, zero-calorie sodas, and calorie-free sweeteners.

The intervention delivery consists of one-on-one telephone counseling by a health coach and an SMS text messaging system to support this target. Participants are supported through telephone counseling during the initial phase (weeks 1-3) of the interventional period and gradually transition to activities that promote self-reliance for behavioral maintenance from week 4 until the end of the study. The intervention incorporates a number of theory-based behavior change strategies in its design [31-33], including those grounded in social cognitive theory and its central tenet of self-efficacy [31].

### SMS Text Messaging System

An SMS text messaging system was developed in collaboration with Mosio, Inc, and is introduced to participants during week 3 of the study for the purpose of self-monitoring, tracking adherence, and encouraging maintenance of prolonged nightly fasting. Participants are asked to text the study team to indicate when they began and ended their overnight fast. Participants receive an automated response to their SMS text messages, with positive reinforcement if the fasting goal was successfully achieved or corrective tools, such as a behavioral strategy, that may help them achieve a longer fast. The messages sent back to participants also contain feedback about their cumulative adherence to the prolonged overnight fasting pattern, calculated as the percentage of the successful overnight fasts during the past week. Participants also receive an SMS text message each day to remind them of their target end time of the overnight fast as well as encouraging SMS text messages to promote adherence that are customized to past performance. An example of SMS text messages sent to and received from participants through the automated SMS text messaging system is provided in Table 1.

**Table 1:** Example of an interaction in which a participant in the intervention arm submits SMS text messages to indicate the beginning and end of overnight fast and, in turn, is provided with automated, encouraging messages promoting cumulative adherence and achievement of the fasting goal.

Time stamp	Message status	Message content
February 17, 2013, 6:00 PM	Sent	Remember: Calories break a fast! Once you start, you need be calorie-free 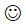
February 17, 2013, 7:28 PM	Received	FAST
February 17, 2013, 7:29 PM	Response	Thanks! You are now running calorie free! Check back in the morning for your end time.
February 18, 2013, 7:00 AM	Sent	Your fast ends at 9.28 am. You can do it!
February 18, 2013, 9:55 AM	Received	EAT
February 17, 2013, 10:05 AM	Response	Congratulations! Your success rate is 100%. Nice work!

### Healthy Lifestyle Education Control

Participants in the control arm receive educational information regarding healthy lifestyle to enhance retention of participants randomized to this group. At baseline, control participants receive an educational session with the health coach and a workbook containing brief information about topics about a healthy lifestyle (eg, sun safety, sleep, hydration, and sitting less). It is notable that research indicates that a single education session has only modest and temporary impacts on behaviors among most individuals [34,35]. To support sustained engagement throughout the 4-month study period, control group participants also receive 1 email and 1 SMS text message per week by the same SMS text messaging system used for participants in the interventional arm. These SMS text messages contain a mixture of educational information and potential personalized touch points to help participants feel valued.

### Primary End Points

A challenge in assessing the clinical benefit of short-term interventions in MGUS, SMM, and SWM is that precursor patients are expected to have a relatively small number of progression events over months to years, limiting the ability to evaluate these events as the outcome. In this study, we focus on body composition, bone marrow adipose tissue, and biomarkers of tumor burden, which are accessible outcome measures in evaluating the efficacy of our lifestyle intervention.

#### Body Composition and Bone Marrow Adiposity

Most investigations evaluating the association of obesity and MGUS and MM have used BMI as a surrogate measure of obesity [12,13,36,37], and although BMI is a convenient, inexpensive measurement [38], it has low specificity for identifying excess adipose tissue and does not account for the type and distribution of fat (visceral and subcutaneous fat) [39,40]. These shortcomings of BMI limit our understanding of the relationship between obesity and the development of MM, as different adipose tissue compartments are known to have differential influences on obesity-related diseases [41-43]. For example, visceral fat is a metabolically active tissue type that releases fatty acids and proinflammatory substances and, in comparison with subcutaneous tissue, is more strongly associated with components of metabolic syndrome [41-43]. Thus, in our study, DXA scan (Horizon W DXA; Hologic, Inc) is used to measure body composition, differentiating not only fat mass from lean mass but also among adipose tissue types (visceral and subcutaneous fat). Furthermore, bone marrow adipose tissue is an understudied adipose depot with endocrine and paracrine signaling functions linked with the proliferation of nearby MM cells [44]. In our study, total marrow lipid content is quantified in bone marrow aspirate samples. Adipocyte-lipid content is analyzed using histology, flow cytometry, and lipidomics on adipocyte-enriched fractions. By measuring and comparing these adiposity measures between baseline and end of study, our study is able to evaluate whether prolonged nightly fasting possibly improves the body composition profile of participants.

#### Clinically Available Tumor Biomarkers

Serum protein electrophoresis supplemented by immunofixation (SPEP/IFX) and serum-free light chains (SFLCs) are the most commonly used clinical tests used to monitor patients at all stages of the disease continuum (MGUS, SMM, and MM). Monoclonal immunoglobulin (M-protein) and light-chain concentrations, as measured by SPEP/IFX and SFLCs, correlate with overall tumor burden and importantly decrease after treatment with a range of mild and aggressive MM therapies [45-47]. These biomarkers are sensitive and measurable variables of tumor response to treatment [48] and, therefore, serve as clinically relevant disease end points for our lifestyle-related intervention pilot study.

### Other End Points

#### Metabolomics

Metabolomics—the high-throughput identification and quantification of small-molecule metabolites—is the study of metabolic changes in biological systems and provides the small-molecule fingerprints that reflect the complex relationships among diet, lifestyle (obesity), genes, and disease processes [49]. Metabolomics can yield novel insights into pathogenesis and risk of cancer and chronic disease [49,50]. Particularly important categories of metabolites in cancer and metabolic disease are metabolites of glycolysis and the tricarboxylic acid cycle [51,52]. These are of interest to this study because of their critical role in tumor growth and because they have been shown to be related to obesity [53]. Metabolomics-based data of MGUS, SMM, and MM are limited to only a small number of studies, including one by Ludwig et al [54] that identified 25 bone marrow metabolites that differed between 10 patients with MGUS and 10 patients with MM [55-59]. Of all molecular entities in the body (eg, genes, transcripts, proteins, and metabolites), metabolites as the final products of biochemical processing have the closest relationship to expressed phenotype [60]. Thus, we believe that metabolomics offers a unique lens to study progression of MM precursors.

#### Quality of Life

Given the high rates of anxiety and distress document in individuals with MGUS and SMM and lack of evidence-based risk reduction strategies available for preventing disease progression to MM [61,62], we aim to evaluate the psychosocial benefits of offering a low-risk lifestyle intervention to this patient population as an exploratory objective. Quality of life is assessed using the well-validated PROMIS (Patient-Reported Outcomes Measurement Information System) global health survey [63-65]. Cancer worry is assessed using a 4-item scale adapted from Lerman et al [66].

#### Baseline Dietary and Lifestyle Assessment

Participants undergo two 24-hour dietary recalls to assess food and beverage consumption (ideally for 1 weekday and 1 weekend day), which are conducted through the Behavioral Measurement and Interventions Shared Resource at the University of Arizona Cancer Center. Participants also complete a baseline survey querying sociodemographic variables (eg, age, sex, race, ethnicity, highest level of education attained, and annual family income), medical comorbidities (eg, hypertension, hyperlipidemia, and diabetes mellitus), and lifestyle factors (eg, smoking, physical activity, sleep impairment, and disturbance) measured by well-validated instruments [67-72]. Finally, participants are asked to wear an ActiGraph accelerometer (GT3XP-BTLE; ActiGraph Corp) on their wrists for 7 days for a baseline assessment of physical activity, sleep, and circadian rhythm parameters [73-75].

#### Feasibility, Acceptability, Fidelity, and Safety

Feasibility is assessed as participant retention (percent dropout in each arm) and percentage of days participants achieved a ≥14-hour fast. Acceptability is assessed as perceived effectiveness of intervention components, plans to continue to engage in a prolonged nightly fast, elements of intervention liked or disliked, and satisfaction with program delivery and staff. Items are rated on a scale from 1 to 5. Finally, fidelity is assessed as percentage of days participants recorded fasting duration via the SMS text messaging system, percentage of SMS text messages read, and percentage of calls with the health coach.

Safety is monitored and assessed by the number and severity of adverse events, according to the revised National Cancer Institute Common Terminology Criteria for Adverse Events (CTCAE) version 5.0. Individuals with any medical or lifestyle condition (eg, diabetes mellitus) that is deemed to elevate their risk of adverse events from the intervention are excluded from the study (Textbox 1). Participants are informed of expected side effects related to fasting (eg, lightheadedness, headaches, restlessness, irritability, and low blood glucose), and adverse events that vary in nature, intensity, and frequency from what is expected are reported.

### Analysis Plan

The key outcomes evaluated in this trial are changes in body composition (with a focus on visceral and subcutaneous adiposity), M-protein concentrations, and bone marrow adiposity. Changes in these end points are evaluated from baseline to follow-up in the 2 groups using a repeated measures mixed-effects model that accounts for the correlation between baseline and follow-up measures and is generally robust to missing data. The baseline values of the dependent variables (eg, weight and M-protein level) and disease subtype are included as covariates in the regression models. Group-by-time interaction terms are included as fixed effects in the regression model. Model fit is assessed using standard methods.

## Results

The PROFAST Study was funded in June 2022. Participant recruitment commenced in April 2023, after developing and testing the text messaging system. As of July 2023, a total of 6 participants consented to the study. The study is expected to be completed by April 2024, and data analysis and results are expected to be published in the first quarter of 2025.

## Discussion

The PROFAST study provides important preliminary data regarding the impact of prolonged nightly fasting on body composition, bone marrow adipose tissue, and biomarkers of tumor burden (SPEP/IFX and SFLCs) in patients with MM precursor conditions, thereby generating hypotheses on how targeting obesity-related mechanisms of carcinogenesis may help prevent MM development.

Obesity is a well-established, potentially modifiable risk factor of MM [11], and there is accumulating evidence that obesity may also increase the risk of MGUS and its progression to overt MM [12-14]. A study of 3 large prospective cohorts of US-based adults followed over 5 million person-years observed a 17% increase in MM risk per 5 kg/m^2^ increase in BMI [36]. In another study of 7818 patients with MGUS in the US Veterans Health Administration database, being overweight and obese were associated with an increased risk of transformation to MM (hazard ratio [HR] for overweight 1.55, 95% CI 1.16-2.06; HR for obesity 1.98, 95% CI 1.47-2.68) [12]. Concordant with these findings, a more recent analysis of patients with MGUS identified through a population-based screening study in Olmstead County, Minnesota, between 1995 and 2003 observed that having a BMI ≥25 was also associated with increased progression to MM or other plasma cell or lymphoid disorders in univariate analysis (HR 2.14, 95% CI 1.05-4.36) and in a multivariable model accounting for clinical factors [13].

These epidemiological studies are supported by mechanistic evidence that obesity may lead to chronic low-grade inflammation and dysregulation of endogenous growth factors linked to myelomagenesis [76,77], together suggesting that weight control may be an effective MM prevention strategy. Indeed, according to a compelling 2016 consensus statement, an expert panel convened by the International Agency for Research on Cancer concluded that there is sufficient mechanistic evidence that a preventative relationship has been established between the “absence of body fatness” and MM [76]. In obesity, adipose tissue, including adipocytes in the bone marrow, is altered in ways that may promote carcinogenesis, including by creating an unfavorable tumor microenvironment in which MM can engraft and grow [44,78]. Notably, the adipose tissue of obese individuals leads to excess free fatty acids as well as altered levels of proinflammatory cytokines (eg, tumor necrosis factor α and interleukin 6), adipokines (eg, adiponectin and leptin), and metabolic peptide hormones (eg, insulin and insulin-like growth factor 1) [44,78,79]. Dysregulated levels of these biological compounds may fuel tumor initiation and influence the genetic characteristics of MM cells in ways that increase cell proliferation [80], reduce apoptosis [81], and contribute to immune cell evasion [82,83]. The implication of these factors is that weight loss may curb the contribution that excess adiposity-associated chronic inflammation and metabolic dysregulation have on MM development.

Beyond weight loss, the PROFAST study tests the hypothesis that “when we eat,” not just “what or how much we eat,” is relevant to cancer prevention [24,25,84,85]. There is evidence suggesting that chronic exposure to circadian rhythm disturbances may lead to metabolic dysregulation, upregulation of proinflammatory cytokines, and abnormal cell proliferation [21,86]. As food intake contributes to the setting of circadian clock rhythms in peripheral organs, such as by inducing changes in body temperature and through the action of hormones such as insulin and nutrient-sensing enzymatic and nuclear receptor signaling systems [21,87], the synchronization of feeding-fasting patterns with circadian rhythms may be important for preventing chronic diseases [24,26,27]. This hypothesis is supported by a large prospective study of patients with early-stage breast cancer, which observed that a short nightly fasting duration (<13 hours per night), compared with a nightly fasting of ≥13 hours, was associated with an increased risk of breast cancer recurrence [25]. In that same study, each 2-hour increase in the nightly fasting duration was associated with lower hemoglobin A_1c_ levels and a longer duration of nighttime sleep [25]. These findings are consistent with the results of population-based studies [24,26,27], including a large case-control study in Spain that observed that diurnal eating patterns, specifically in maintaining longer time intervals between the last meal of the day and initiation of sleep, were inversely associated with risk of breast and prostate cancer [27]. Similarly, two US-based studies of 2009-2010 National Health and Nutrition Examination Survey data evaluating biomarkers of breast cancer risk observed that a longer duration of nighttime fasting was associated with improved measures of glycemic control and systemic inflammation [24,26], aligning with pathophysiological mechanisms underlying cancer risk [76].

Results from these human observational studies are consistent with rodent studies demonstrating that mice subjected to a time-restricted (16-hour) fasting regimen of a high-fat diet during the sleep phase were protected against weight gain, abnormal glucose metabolism, and inflammation, all of which were associated with cancer outcomes [22,88-91]. Notably, protective effects were observed despite these mice having the same caloric intake as those that had ad libitum access to food and ate frequently throughout day and night [22], suggesting that the beneficial effects of this fasting regimen were partly mediated through mechanisms independent of calorie restriction. Together, these data provide a strong basis of our intervention aimed to curb the inflammatory and metabolic mechanisms shown to contribute to myelomagenesis [44,77].

Finally, and importantly, prolonged nightly fasting represents a lifestyle modification that is safe, practical, and acceptable for patients who otherwise are managed by a “watchful waiting” strategy. Patients with MM precursor conditions are reported to have diminished quality of life, increased comorbidities, and heighted anxiety and a sense of loss of control regarding their MM risk [61,62,92,93]. There is, therefore, a need to identify lifestyle-based interventions that patients can safely and practically adopt into their daily lifestyles and ultimately help curb disease progression. Fasting, as a practice, developed independently among different people groups and religions (eg, Ramadan in Islam) around the world [94], and intermittent fasting has become one of the most common dietary patterns reported in the United States [95]. Prolonged nightly fasting is attractive because of its simplicity and feasibility, as supported by evidence from clinical trials of time-restricted eating strategies [16,23], and, thus, may be a potentially effective disease prevention strategy at the population level [16].

The limitations of this study include the relatively small sample size of the study and the potential lack of sociodemographic and geographic diversity of participants due to recruitment occurring at a single academic institution, thus, possibly impacting generalizability of findings. Also, participants willing to participate in a 4-month prolonged overnight fasting intervention may not be representative of the general population of patients with overweight or obesity with MM precursors, and selection bias for patients who are motivated to participate in PROFAST could increase the study adherence rate. Furthermore, although participants in the intervention group are prescribed solely a prolonged overnight fasting regimen with no other recommendations regarding lifestyle patterns, it is theoretically possible that participants may change their dietary, physical activity, and sleep habits due to paying closer attention to the timing of their eating and evaluations of their body composition before and after the intervention. These changes may also impact outcomes.

In summary, the study described herein serves as an important first step in exploring the premise that prolonged nightly fasting is a practical, effective strategy to control obesity and intercept disease progression in individuals with MM precursor conditions. By evaluating the impact of this lifestyle intervention on relevant biomarkers of excess adiposity and myeloma tumor burden, this pilot study may generate hypotheses and inform further investigations in identifying clinical and public health strategies for MM prevention.

## Abbreviations

CTCAE: National Cancer Institute Common Terminology Criteria for Adverse Events
DXA: dual-energy x-ray absorptiometry
HR: hazard ratio
MGUS: monoclonal gammopathy of undetermined significance
MM: multiple myeloma
PROFAST: PROlonged nightly FASTing
PROMIS: Patient-Reported Outcomes Measurement Information System
SFLC: serum-free light chain
SMM: smoldering multiple myeloma
SPEP/IFX: serum protein electrophoresis supplemented by immunofixation
SWM: smoldering Waldenström macroglobulinemia

## Data Availability

Data sharing is not applicable to this article as no data sets were generated or analyzed during this study.
